# Construction and validation of prognostic signatures related to mitochondria and macrophage polarization in gastric cancer

**DOI:** 10.3389/fonc.2024.1433874

**Published:** 2024-07-26

**Authors:** Yan Zhang, Jian Cao, Zhen Yuan, Hao Zuo, Jiacong Yao, Xiaodie Tu, Xinhua Gu

**Affiliations:** ^1^ Department of Gastrointestinal Surgery, Suzhou Municipal Hospital, Affiliated Suzhou Hospital of Nanjing Medical University, Gusu School of Nanjing Medical University, Suzhou, China; ^2^ Department of Gastroenterology, Suzhou Municipal Hospital, Affiliated Suzhou Hospital of Nanjing Medical University, Gusu School of Nanjing Medical University, Suzhou, China; ^3^ Alliance Biotechnology Company, Hangzhou, China

**Keywords:** gastric cancer, mitochondria, macrophage polarization, single-cell data, prognostic signature

## Abstract

**Background:**

Increasing evidence reveals the involvement of mitochondria and macrophage polarisation in tumourigenesis and progression. This study aimed to establish mitochondria and macrophage polarisation-associated molecular signatures to predict prognosis in gastric cancer (GC) by single-cell and transcriptional data.

**Methods:**

Initially, candidate genes associated with mitochondria and macrophage polarisation were identified by differential expression analysis and weighted gene co-expression network analysis. Subsequently, candidate genes were incorporated in univariateCox analysis and LASSO to acquire prognostic genes in GC, and risk model was created. Furthermore, independent prognostic indicators were screened by combining risk score with clinical characteristics, and a nomogram was created to forecast survival in GC patients. Further, in single-cell data analysis, cell clusters and cell subpopulations were yielded, followed by the completion of pseudo-time analysis. Furthermore, a more comprehensive immunological analysis was executed to uncover the relationship between GC and immunological characteristics. Ultimately, expression level of prognostic genes was validated through public datasets and qRT-PCR.

**Results:**

A risk model including six prognostic genes (GPX3, GJA1, VCAN, RGS2, LOX, and CTHRC1) associated with mitochondria and macrophage polarisation was developed, which was efficient in forecasting the survival of GC patients. The GC patients were categorized into high-/low-risk subgroups in accordance with median risk score, with the high-risk subgroup having lower survival rates. Afterwards, a nomogram incorporating risk score and age was generated, and it had significant predictive value for predicting GC survival with higher predictive accuracy than risk model. Immunological analyses revealed showed higher levels of M2 macrophage infiltration in high-risk subgroup and the strongest positive correlation between risk score and M2 macrophages. Besides, further analyses demonstrated a better outcome for immunotherapy in low-risk patients. In single-cell and pseudo-time analyses, stromal cells were identified as key cells, and a relatively complete developmental trajectory existed for stromal C1 in three subclasses. Ultimately, expression analysis revealed that the expression trend of RGS2, GJA1, GPX3, and VCAN was consistent with the results of the TCGA-GC dataset.

**Conclusion:**

Our findings demonstrated that a novel prognostic model constructed in accordance with six prognostic genes might facilitate the improvement of personalised prognosis and treatment of GC patients.

## 1 Introduction

Gastric cancer is one of the most common malignancies in the world. Gastric cancer is a complex disease, and its etiology is not fully understood at present. Helicobacter pylori infection, environmental factors, genetic factors, etc. may be related to the occurrence of gastric cancer. About 950,000 new cases of gastric cancer are reported every year around the world, with nearly 700,000 deaths (1). At present, there are still many deficiencies in the diagnosis and treatment of gastric cancer. Due to the unobvious early symptoms of gastric cancer, many patients are in the middle and late stages when they are found, which greatly reduces the success rate of treatment. At present, laparoscopic surgery, and even robotic surgery, have been widely used in the treatment of gastric cancer patients, which is the most important treatment method for gastric cancer. However, due to the physical condition, disease progression and other reasons, surgical treatment often cannot achieve radical results (2). In addition, there are great limitations in the chemotherapy of gastric cancer. The selectivity of chemotherapy drugs is not strong, which may cause damage to normal cells of patients and produce side effects. For gastric cancer patients who have metastasis, the effect of chemotherapy is not ideal (3). Although targeted therapy and immunotherapy for gastric cancer have made some progress, they are still in the exploratory stage, and their efficacy and safety need further verification and improvement (4). Currently, liquid biopsy is playing an increasingly important role in the diagnosis and treatment of gastric cancer (5). It is necessary to find more reliable biomarkers to predict the prognosis of gastric cancer and explore more potential therapeutic targets.

Mitochondrial function and macrophage polarization processes are associated with a variety of tumors, including gastric cancer. Mitochondria are important organelles in cells that can participate in the metabolism of various substances, including carbohydrates, fats, and proteins. Mitochondria maintain the normal physiological functions of cells through oxidative phosphorylation. Abnormal mitochondrial function may lead to metabolic abnormalities in the body. Tumor cells often have various abnormalities in mitochondrial function, such as changes in mitochondrial metabolic pathways and dysregulation of mitochondrial autophagy. These abnormalities can lead to disturbed energy metabolism and uncontrolled growth of tumor cells (6). Mitochondria contain mitochondrial DNA (mtDNA), which is the genetic material in mitochondria and is double-stranded and circular. mtDNA carries its own genetic information, including 37 genes, which encode certain proteins and RNAs within the mitochondria. Many studies have found mitochondrial DNA mutations in various malignant tumors. Abnormal proteins produced by mutated mitochondrial DNA can not only help tumor cells proliferate, but also enable them to migrate and invade distal organs (7). Abnormal copies of mitochondrial genes are often associated with poor prognosis for patients (8). In addition to mtDNA, there are also a large number of mitochondrial-related genes in the genome, which are closely related to mitochondrial function. Mutations in these genes often cause abnormal mitochondrial function in tumor cells (9). Therefore, further research on mitochondria may not only help us understand the physiological and pathological processes of various tumors such as gastric cancer, but also provide new ideas and methods for cancer treatment.

Macrophage polarization refers to the process that macrophages exhibit different functional and phenotypic characteristics under different stimuli. It is a complex cellular process that involves various signaling pathways and molecular regulatory mechanisms (10). Macrophages can be divided into M1 macrophages and M2 macrophages, and can be further divided into various subtypes. Macrophage polarization is a complex process involving various regulatory mechanisms, including inflammatory factors and anti-inflammatory factors, as well as various genes involved in the process of macrophage polarization (11, 12). Currently, macrophage polarization-related genes have been successively discovered, such as IRF5 and STAT1, which activate innate immune responses by inducing the expression of cytokines (13); STAT3 plays an important role in controlling macrophage proliferation and differentiation (14). Macrophage polarization also plays an important role in tumors. In the tumor microenvironment, M1 macrophages have the characteristics of killing tumor cells, producing inflammatory factors and anti-tumor immune responses, while M2 macrophages are more involved in tissue repair and immunosuppression (15). Studies have shown that in gastric cancer tissue, the number of M2 macrophages is significantly increased, while the number of M1 macrophages is decreased. This polarization imbalance can promote tumor growth and immunosuppression, providing favorable conditions for the development of gastric cancer (16). Cytokines, growth factors, and chemical substances released by tumor cells, as well as abnormal tumor microenvironments, can regulate the process of macrophage polarization. These factors can affect the signal transduction pathway of macrophages, thereby changing the function and polarization state of macrophages (17). Therefore, regulating tumor-associated macrophage polarization may become a new strategy for tumor treatment. Some drugs can inhibit the polarization of M2 macrophages, thereby enhancing the anti-tumor function of macrophages (18).

There is a close relationship between mitochondrial dysfunction and macrophage polarization in tumors. Mitochondrial dysfunction can promote the polarization of tumor-associated macrophages (TAMs). Mitochondrial damage-associated molecular patterns (DAMPs) released by tumor cells can activate macrophages and induce their polarization towards pro-inflammatory (M1) or anti-inflammatory (M2) phenotypes (19). Macrophage polarization can also affect mitochondrial function in tumor cells. For example, M1-type TAMs can release reactive oxygen species (ROS) and other oxidative stress molecules, leading to mitochondrial damage and energy metabolism disorders in tumor cells; while M2-type TAMs can secrete growth factors and anti-inflammatory factors to promote tumor cell growth and survival (20). In summary, there is an interactive and regulatory relationship between mitochondrial dysfunction and macrophage polarization in tumors. Mitochondrial dysfunction can promote the polarization of TAMs, while macrophage polarization can also affect mitochondrial function and biological behavior of tumor cells.

Currently, there are still limited reports on the relationship between mitochondrial and macrophage polarization-related genes in tumors. There is even less literature on prognostic genes related to these two functions and their underlying molecular mechanisms in gastric cancer. This study identified prognostic genes related to mitochondrial and macrophage polarization in gastric cancer patients based on public database data, including transcriptome data and single-cell data, and constructed a prognostic model. In addition, based on the prognostic model, we further explored the biological pathways involved in prognostic genes and their relationships with clinical features and tumor immune microenvironment. In summary, this study identified prognostic genes related to mitochondrial and macrophage polarization in gastric cancer and validated them in clinical samples. By exploring the key genes underlying the intrinsic relationship between the two, we provide a new perspective for understanding the pathogenesis and development of gastric cancer, and also provide new ideas and methods for tumor treatment.

## 2 Materials and methods

### 2.1 Data collection

TCGA database provided the mRNA expression profiles and accompanying clinical data of 375 stomach adenocarcinoma tumor tissue samples (GC samples) and 32 paraneoplastic tissue samples (normal samples), and this set of data was referred to as the TCGA-GC dataset. Meanwhile, GEO database (http://www.ncbi.nlm.nih.gov/geo/) provided GC-related original microarray data, specifically, the GSE15459 dataset with 191 GC samples, and the GSE13911 dataset which contained 38 GC samples and 31 normal samples, as well as both datasets were based on the GPL570 platform (21, 22). Similarly, the GSE183904 dataset comprised high-throughput sequencing data from 26 GC tissue samples, 10 normal tissue samples and four peritonium tissue samples for single-cell data analysis (23). There was no significant statistical difference in basic information such as the age and gender of patients in the above-mentioned datasets. Furthermore, a total of 1,136 mitochondria-related genes (MRGs) (**Supplementary Table 1** **)** and 35 macrophage polarization-related genes (MPRGs) (**Supplementary Table 2** **)** were collected by accessing MitoCarta3.0 database (https://www.broadinstitute.org/mitocarta) and Molecular Signatures Database (MsigDB, http://www.broadinstitute.org/gsea/msigdb/index.jsp), respectively.

### 2.2 Differential expression analysis

In order to acquire the differentially expressed genes 1 (DEGs1) between GC and normal groups in single-cell sequencing data (GSE183904), the ‘FindMarker’-function divided in ‘Seurat’-package (v 4.3.0) (24) was utilized to carry out differential expression analysis, and the screening condition was adj*.P*<0.05. Meanwhile, DEGs2 between GC and normal groups in TCGA-GC were identified via ‘DESeq2’-package (v 1.36.0) (25), with the filtering conditions of adj*.P*<0.05 and |log_2_FoldChange(FC)|>0.5. The ‘ggplot2’-package (v 3.4.1) (26) and ‘ComplexHeatmap’-package (v 2.12.1) (27) were utilized to create the volcano map and heat map of DEGs2, respectively.

### 2.3 Weighted gene co-expression network analysis(WGCNA)

In our study, based on MRGs and MPRGs as background gene sets, the MRGs score and MPRGs score for each sample of TCGA-GC were calculated via ‘GSVA’-package (v 1.38.2) (28), followed by a rank-sum test to compare the differences in MPRGs score and MRGs score between GC and normal groups (*P*<0.05). Subsequently, depending on the expression data of the GC samples in TCGA-GC dataset, WGCNA was implemented via the ‘WGCNA’-package (v 1.72–1) (29) to identify the module and module genes that were most relevant to the MRGs score and MRGs score. To begin with, the GC samples were clustered and outlier samples were removed to determine the accuracy of subsequent analyses. Next, the optimal soft threshold was determined at R^2^ crossing the threshold 0.80 (red line) and mean connectivity also tending to 0 for ensuring that interactions between genes maximally matched the scale-free distribution. The systematic clustering tree was obtained by utilizing the adjacency connection and gene similarity, and following that, the co-expression network was constructed according to the guidelines of the hybrid dynamic tree cutting algorithm (minModuleSize=50 and mergeCutHeight=0.5). Ultimately, a module-trait heatmap was created to further determine the key module in GC that was most significant with MRGs score and MPRGs score by comparing the correlation coefficient and *P*-value (*P*<0.05). The genes contained in key module were defined as key module genes highly correlated with the MRGs score and MPRGs score.

### 2.4 Screening of candidate genes and functional annotation analysis

The intersections of DEGs1, DEGs2, and key module genes were taken utilizing ‘ggVennDiagram’-package (v 1.2.2) (30), and the intersecting genes were called candidate genes for follow-up analysis. Furthermore, in order to further reveal the biological functions exerted by the candidate genes, enrichment analysis was undertaken. Specifically, enrichment analyses on the basis of Gene Ontology (GO) and Kyoto Encyclopedia of Genes and Genomes (KEGG) databases were implemented via ‘clusterProfiler’-package (v 4.4.4) (31) and ‘org.Hs.eg.db’-package (v 3.15.0) (32) with a significance of *P*<0.05.

### 2.5 Creation and validation of risk model

To begin with, the ‘survival’-package (v 3.3–1) (33) was applied to carry out univariate Cox regression analysis on the basis of candidate gene expression in 351 GC samples with survival data from TCGA-GC dataset, and prognosis-related genes in GC were acquired with HR≠1 and *P*<0.05 as filter conditions. Subsequently, prognosis-related genes that passed the PH test (*P*>0.05) were subject to LASSO analysis via ‘glmnet’-package (v 4.1–6) (34), followed by identifying prognostic genes in GC based on lambda_min_ value. What’s more, risk model was created, and the risk score of GC patient was computed on the basis of the expression levels of prognostic genes and their coefficients with the following formula:


$$risk\;score=\sum_{i=1}^{n}\left(Coefi*Expi\right)$$

In TCGA-GC dataset, the GC sample was classified into two risk subgroups (high- and low-risk subgroups) in accordance with median risk score. Next, analyses of risk curves, survival status, and prognostic signature gene expression were completed depending on survival and expression data of samples in two risk subgroups. Following this, Kaplan-Meier (KM) survival analysis was achieved via ‘survminer’-package (v 0.4.9) with the aim of comparing the survival differences (*P*<0.05) between the two risk subgroups. Meanwhile, receiver operating characteristic (ROC) curves at 1-, 3- and 5-years were displayed utilizing ‘survivalROC’-package (v 1.0.3) (35), and the precision of risk model in forecasting the prognosis of GC on the basis of area under curve (AUC) value was evaluated. In general, AUC value greater than 0.6 indicated favorable performance of the risk model. Ultimately, the same approaches were employed in GSE15459 dataset to validate the generalisability of the risk model to predict GC prognosis.

### 2.6 Clinical correlation analysis

According to 351 GC samples with clinical data in TCGA-GC dataset, the number of patients with different clinical subgroups was compared between high- and low-risk subgroups with the use of the chi-square test to explore the association between risk score and GC clinical characteristics. Specifically, the clinical characteristics included age, gender, vital status, overall survival (OS), pathologic-M/N/T, pathologic-stage and grade. Subsequently, based on the clinical characteristics associated with the risk scores, comparison of risk scores between different clinical subgroups was undertaken by rank sum test (two subgroups) and ANOVA test (three and more subgroups), followed by visualization of the results using box plots and Sankey diagrams. Eventually, the expression level analysis of prognostic genes were achieved between different clinical subgroups.

### 2.7 Independent prognostic analysis and nomogram creation

By combining risk score with seven conventional clinical characteristics (age, gender, grade, pathologic-M/N/T, and pathologic-stage), univariate and multivariate Cox regression analyses (*P*<0.05) as well as proportional hazard (PH) hypothesis test (*P*>0.05) were performed in the TCGA-GC dataset to further assess the possibility of utilizing them as independent prognostic indicators for GC. After selecting the independent prognostic indicators, we created a nomogram of 1, 3, and 5-year survival via ‘rms’-package (v 6.5–0) (36). What’s more, the ROC and calibration curves were generated to determine the predictive efficacy of this nomogram.

### 2.8 Gene set enrichment analysis (GSEA)

GSEA was accomplished with the aim of uncovering biological pathway differences between the two risk subgroups. To begin with, differential expression analysis was performed between two risk subgroups in TCGA-GC dataset, and DEGs were sorted in accordance with log2FC. Next, based on the KEGG database, GSEA-KEGG was carried out on the sorted genes via ‘clusterProfiler’-package (v 4.4.4) (31), with thresholds of adj*.P*<0.05 and |NES|>1.

### 2.9 Tumor immune microenvironment (TIME) analysis

In an attempt to elucidate the association between risk score and immunological characteristics, a more comprehensive immunological analysis was accomplished. Firstly, the abundance of individual immune cells for each sample was obtained through calculating the scores of 22 immune cells in TCGA-GC dataset utilizing CIBERSORT algorithm, followed by a rank-sum test to analyze the differences in immune cell scores between two risk subgroups (*P*<0.05). Subsequently, Spearman correlation analysis was executed between risk score and differential immune cells as well as between prognostic genes and differential immune cells, respectively, so as to uncover the relationship between them.

Furthermore, immune checkpoint genes (ICGs) play a critical role in the TIME. Therefore, this work compared the ICG expression discrepancy between two risk subgroups on the basis of 43 ICGs obtained from published literature (37). The Spearman correlation analysis between prognostic genes and differential ICGs was also performed. What’s more, tumor immune dysfunction and exclusion (TIDE) score was calculated for each GC sample in TCGA-GC dataset through accessing TIDE database (http://tide.dfci.harvard.edu), and differences between two risk subgroups were compared by a rank-sum test to predict treatment response to immune checkpoint inhibitors.

Meanwhile, immunity, stromal, and ESTIMATE scores were computed through ESTIMATE method for all GC samples from TCGA-GC dataset with the aim of comparing the differences in each score between two risk subgroups (P<0.05).

### 2.10 Chemotherapy drug sensitivity, MSI, TMB, and CNV analyses

In TCGA-GC dataset, the 50% inhibitory concentration (IC50) values of 138 chemotherapeutic drugs for each GC sample were obtained via the ‘pRRophetic’-package (v 0.5) (38), and these 138 agents were retrieved from genomics of drug sensitivity in cancer (GDSC) database (https://www.cancerrxgene.org/). Next, IC50 values of 138 chemotherapeutic agents were subjected to the rank-sum test to identify agents that differed markedly between two risk subgroups. Meanwhile, the fold change (FC) was calculated, followed by classifying the agents into three groups based on *P* value and FC, namely sensitive low-risk (*P*<0.05 and FC>0.2), sensitive high-risk (*P*<0.05 and FC<-0.2), and no sensitive. Subsequently, the top five agents of sensitive high-risk/sensitive low-risk were selected for correlation analysis with the risk score.

In the meantime, on the basis of microsatellite instability (MSI) data in GC patients gained from cBiPortal database (https://www.cbioportal.org/), GC samples with MSI score>0.3 were defined as the MSI group, and GC samples with MSI score<0.3 were defined as the MSS (microsatellite stable) group in TCGA-GC dataset. Subsequently, the difference in risk score between MSI and MSS groups was compared, and Spearman correlation analysis was performed between MSI score and risk score. More importantly, the top five agents of sensitive high-risk/sensitive low-risk were selected for correlation analysis with MSI score.

Additionally, tumor mutation burden (TMB) and copy number variation (CNV) data for GC samples were derived from the cBiPortal and UCSC Xena (https://xena.ucsc.edu/) databases, respectively. Next, the differences in TMB and CNV between two risk subgroups were compared via rank-sum test (*P*<0.05), followed by Spearman correlation analysis between them and risk score.

### 2.11 Single-cell data analysis and pseudo-time analysis

Single-cell sequencing data (GSE183904) were imported into R software and analyzed using the ‘Seurat’-package (v 4.3.0) (24). Initially, the data were filtered using the ‘CreateSeuratObject’-function with the following filtering criteria: (1) genes expressed in fewer than 3 cells were eliminated; (2) cells with a total gene count greater than 200 were retained. Secondly, the percentage of mitochondrial genes was computed via ‘PercentageFeatureSet’-function, and cells with a percentage less than 10% were retained to ensure that low quality cells were excluded. For downstream analysis, data were normalized utilizing ‘NormalizeData’-function, and ‘FindVariableFeatures’-function was adopted to identify top 2,000 highly variable genes after quality control (QC) with the ‘vst’ method. Immediately thereafter, multiple samples were combined and canonical correlation analysis was implemented to remove batch effects. Based on these 2,000 genes, principal component analysis (PCA) was implemented for dimensionality reduction, and then the cells were clustered using ‘FindNeighbors’ and ‘FindClusters’-functions to yield cell clusters. Additionally, ‘FindAllMarkers’-function was applied to discover the significant marker genes for each cell cluster by setting the parameters min.pct=0.25, only.pos=TRUE, and logfc.threshold=0.7. What’s more, to identify cell subpopulations, cell clusters were annotated according to the CellMarker database (http://biocc.hrbmu.edu.cn/CellMarker/), and marker genes in different cell subpopulations were displayed.

Further, the expression level of prognostic genes in each cell subpopulation was demonstrated, and cell subpopulation with higher expression of prognostic genes and with expression of each gene was utilized as the key cell for subsequent analyses. Immediately thereafter, the identified key cell was analyzed for functional enrichment via ‘ReactomeGSA’-package (v 1.4.2) (39). Eventually, based on key cells, unsupervised cluster analysis was implemented via ‘FindNeighbors’ and ‘FindClusters’ functions to identify subclasses of key cells, followed by pseudo-time analysis of these subclasses via ‘monocle3’-package (v 1.0.0) (40).

### 2.12 Chromosomal localization and subcellular localization analyses

The subcellular localization analysis was performed using the ‘RCircos’-package (v 1.2.2) (41) for determining the location of prognostic genes on human chromosomes. Simultaneously, the function of prognostic genes was closely linked to their location in the cell, so it was important to know the subcellular location of these genes. In this study, the subcellular localization of prognostic genes was predicted by visiting the online website mRNALocater (http://bio-bigdatacn/mRNALocater).

### 2.13 Regulatory network analysis

To uncover the molecular regulatory mechanisms of prognostic genes in GC, lncRNA-miRNA-mRNA regulatory network was established. Initially, miRWalk database(http://mirwalk.umm.uni-heidelberg.de/) was employed to forecast miRNAs that could target prognostic genes. Later on, upstream lncRNAs of miRNAs were predicted by accessing starBase database (http://starbase.sysu.edu.cn/), with the filtering conditions of clipExpNum≥2, degraExpNum≥2, and pancancerNum≥2. The lncRNA-miRNA-mRNA network was generated with the help of Cytoscape software (v 3.9.1) (42).

### 2.14 Expression level analysis and validation

In TCGA-GC and GSE13911 datasets, expression level of prognostic genes in GC and normal samples was analyzed. Subsequently, expression analysis of prognostic genes was finished through quantitative real time polymerase chain reaction (qRT-PCR). Ten clinical gastric tissue samples were collected from Suzhou Municipal Hospital, Affiliated Suzhou Hospital of Nanjing Medical University, including five GC patients and five paraneoplastic patients. The experiment was approved by the Ethics Committee of Gusu School, Nanjing Medical University.

Total RNA was isolated from gastric tissue using TRIzol reagent, followed by reverse transcription to synthesize complementary DNA (cDNA) using SweScript First Strand cDNA synthesis kit. The qRT-PCR was run for 40 cycles under the following conditions, 95°C for 1 min, 95°C for 20 s, 55°C for 20 s, and 72°C for 30 s. GAPDH served as the internal reference gene for biomarkers, and the relative expression levels of prognostic genes were quantified using the 2^-ΔΔCT^ method. Primers were shown in **Table 1**.

**Table 1:** Primers used in PCR experiments.

Gene	Forward Primer	Reverse Primer
RGS2	ATTCAGCCTGGGTGTTCAGG	AGACACCACGTTCAGACCAC
GJA1	CAGCCACTAGCCATTGTGGA	GGCTGTTGAGTACCACCTCC
GPX3	AGAAGTCGAAGATGGACTGCC	GGGAAAGCCCAGAATGACCA
LOX	GTGGGCGAAGGTACAGCATA	TGACAACTGTGCCATTCCCA
VCAN	TCGAGGAGGCTGCAAAAGAG	TGCAGCGATCAGGTCGTTTA
CTHRC1	GGGAGGTGGTGGACCTGTAT	GTCCTTCCACGCAATTTTCC
GAPDH	CGAAGGTGGAGTCAACGGATTT	ATGGGTGGAATCATATTGGAAC

### 2.15 Statistical analysis

In the present work, all statistical analyses involved were performed by R program (v 4.2.1). Discrepancies between groups were completed by rank sum test (two subgroups) or ANOVA test (three and more subgroups). *P-*value<0.05 was deemed statistically meaningful unless otherwise stated.

## 3 Results

### 3.1 Key module genes correlated with MRGs and MPRGs scores were obtained through WGCNA

**Figure 1:**
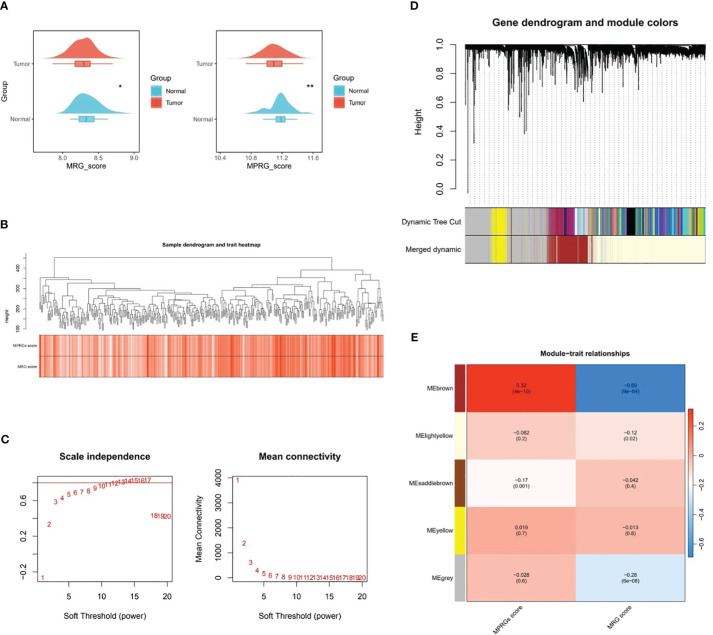
Key modular genes correlated with MRGs and MPRGs scores obtained through WGCNA. **(A)** MRGs and MPRGs scores between gastric cancer (GC) group and normal group in TCGA-GC dataset, **(B)** Sample dendrogram and trait heatmap, **(C)** Scale independence and mean connectivity among genes, **(D)** Gene dendrogram and module colors, **(E)** Trait relationships of different colored modules. * indicates p<0.05, ** indicates p<0.01.

In TCGA-GC dataset, there were remarkable differences in MRGs score and MPRGs score between GC and normal groups (*P*<0.05), and both MRGs and MPRGs scores were lower in GC samples than in normal samples (**Figure 1A**). Subsequently, WGCNA was implemented to excavate the modules and module genes that related to MRGs score and MPRGs score. After the cluster analysis of the samples, no outlier samples were observed (**Figure 1B**). Also, the soft threshold of 14 was chosen to construct the co-expression network, at which point interactions between genes maximally matched the scale-free distribution (**Figure 1C**). In the process of constructing the co-expression network, five modules were created with the systematic clustering tree and dynamic tree cutting algorithms (**Figures 1D, E** **)**. The module MEbrown demonstrated the strongest association with MRGs score and MPRGs score, with a negative correlation with MRGs score (cor=-0.69 and *P*<0.001) and a positive association with MPRGs score (cor=0.32 and *P*<0.001). Ultimately, a total of 3,110 genes contained in module MEbrown were identified as key module genes highly linked to MRGs score and MPRGs score.

### 3.2 Candidate genes were strongly associated with immune responses and cytokines

**Figure 2:**
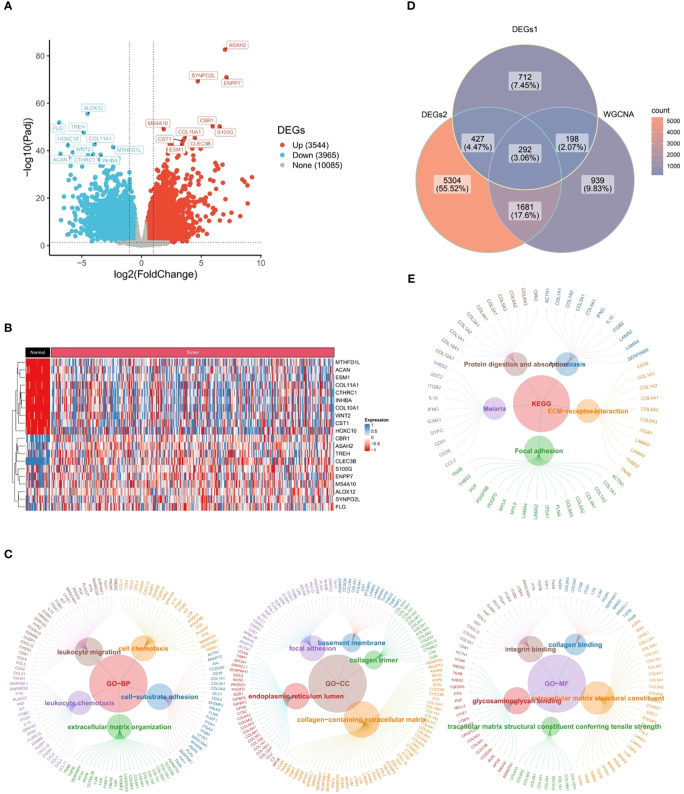
Candidate genes closely related to immune response and cytokines. **(A)** 1,628 differentially expressed genes (DEGs1) in GSE183904 dataset, **(B)** 7,704 differentially expressed genes (DEGs2) in TCGA-GC dataset, **(C)** Venn diagram obtained by intersecting DEGs1, DEGs2, and key modular genes, **(D)** Go functional enrichment analysis of candidate genes, **(E)** KEGG enrichment analysis of candidate genes.

Through differential expression analysis, a total of 1,628 DEGs1 in GSE183904 dataset and 7,704 DEGs2 (3,625 up-regulated and 4,079 down-regulated) in TCGA-GC dataset were mined (**Figures 2A, B**). Subsequently, the Venn diagram demonstrated that 292 candidate genes were acquired through fetching the intersections of 1,628 DEGs1, 7,704 DEGs2 and 3,110 key module genes (**Figure 2C**).

Further, 292 candidate genes were significantly enriched into 800 GO items [699 biological processes (BPs), 60 cellular components (CCs), and 41 molecular functions (MFs)] and 14 KEGG pathways (*P*<0.05). The important GO-BP categories were primarily connected with immune responses and cytokines, such as “leukocyte migration”, “leukocyte mediated immunity”, “cell chemotaxis”, “leukocyte activation involved in immune response”, etc (**Figure 2D**). The candidate genes were highly enriched in the “endoplasmic reticulum lumen”, “collagen trimer” and “basement membrane” in GO-CC analysis (**Figure 2D**). In GO-MF category, they showed concentration in “growth factor binding”, “immunoglobulin binding”, “transforming growth factor beta binding”, etc (**Figure 2D**). Meanwhile, KEGG analysis elucidated that candidate genes were engaged in “PI3K-Akt signaling pathway”, “Phagosome”, “ECM-receptor interaction”, and other signaling pathways (**Figure 2E**). These findings strongly revealed that immune responses and cytokines were highly relevant to the pathogenesis and progression of GC.

### 3.3 Risk model was effective in predicting prognosis of GC

**Figure 3:**
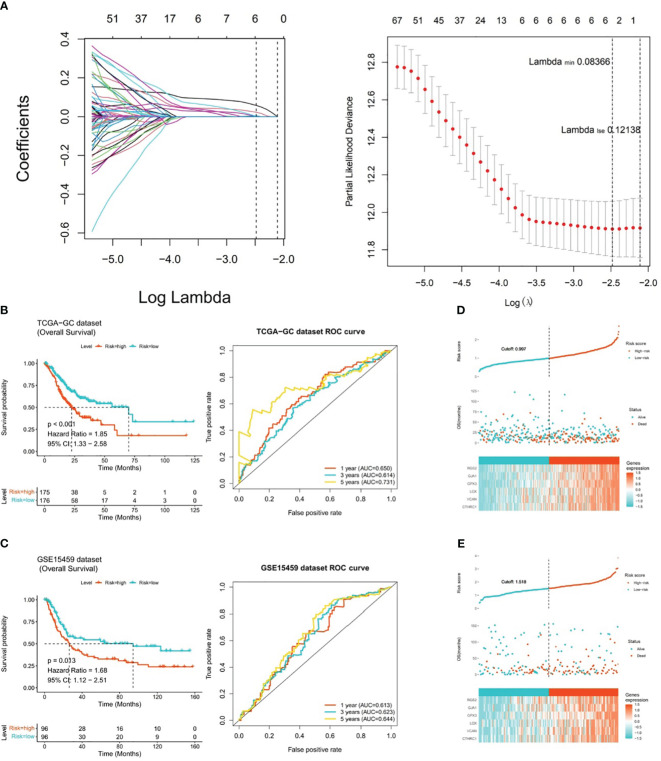
Risk model for predicting the prognosis of gastric cancer. **(A)** Six prognostic genes (GPX3, GJA1, VCAN, RGS2, LOX, and CTHRC1) selected by LASSO regression analysis to construct the risk model, **(B)** K-M curve and ROC analysis between high-risk and low-risk patients in TCGA-GC dataset (P<0.001), **(C)** K-M curve and ROC analysis between high-risk and low-risk patients in GSE15459 dataset (P=0.013), **(D)** Heatmap of the expression of six prognostic genes in patients from TCGA-GC dataset, **(E)** Heatmap of the expression of six prognostic genes in patients from GSE15459 dataset.

After incorporating 292 candidate genes into univariate Cox regression analysis and PH hypothesis test, totally 101 genes were identified that were significantly associated with prognosis in TCGA-GC dataset (**Supplementary Figure 1**). Immediately, with respect to the LASSO regression analysis, the model was optimal when lambda_min_ was equal to 0.08366, and six prognostic genes (GPX3, GJA1, VCAN, RGS2, LOX, and CTHRC1) were chosen to create risk model (**Figure 3A**). On the basis of the coefficients of these six genes in LASSO analysis, we computed risk score by following the formula below: Risk score = GPX3*0.145 + GJA1*0.071 + VCAN*0.005 + RGS2*0.072 + LOX*(-0.025) + CTHRC1*0.110. In TCGA-GC dataset, the GC patients were categorized into two risk subgroups (low- and high-risk subgroups) in accordance with median risk score. As demonstrated in **Figure 3B**, the K-M curve revealed that the survival rate of high-risk patients was markedly lower than that of low-risk patients (*P*<0.001). In ROC analysis, AUC values for 1-, 3-, and 5-year were 0.650, 0.614, and 0.731, correspondingly, which implied that risk model was stable and effective in forecasting the prognosis of GC patients (**Figure 3B**). In order to evaluate the robust prediction value of risk model, these were additionally further validated in GSE15459 dataset. The results indicated that the significant prognostic value was *P*=0.013, and AUC values at 1-, 3-, and 5-year survival were 0.613, 0.623, and 0.644, correspondingly (**Figure 3C**). The relationships of risk score with survival time and survival status, as well as the heat map of expressions of the six prognostic genes, were illustrated in **Figures 3D, E**. Obviously, with increasing risk score, the survival time of the patient decreased and the number of deaths rose at the same time.

### 3.4 Association analysis of risk score with GC clinical characteristics

**Figure 4:**
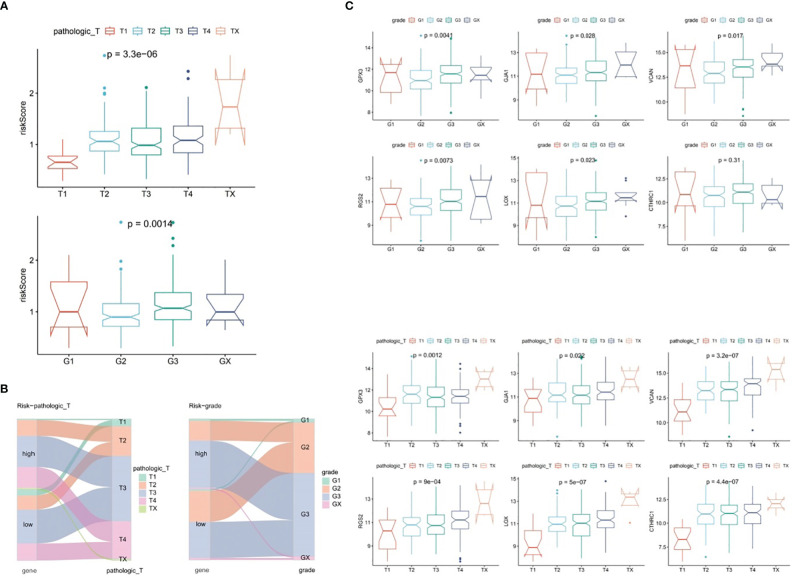
Correlation analysis of risk scores with clinical characteristics of gastric cancer in the TCGA-GC dataset. **(A)** Differences in risk scores among patients with different pathological T stages and grades, **(B)** Representation of patient flow into different subgroups through Sankey diagram, **(C)** Expression profiles of six prognostic genes between patients with pathological T1 to TX stages and grades 1 to X.

In TCGA-GC dataset, the relationship between risk score and various clinical characteristics was further investigated to reveal the effect of risk score in GC progression. Firstly, clinical characteristics of the two risk subgroups were compared, and the differences in vital status (*P*=0.001), OS (*P*=0.029), pathologic-T (*P*=0.007) and grade (*P*=0.017) between two risk subgroups were statistically significant (**Table 2**). Among them, there were marked discrepancies in risk scores of patients with different pathologic-T and grade, and pathologic-TX and grade-3 were associated with higher risk score (**Figure 4A**). Meanwhile, the Sankey plot also demonstrated that the majority of T1 and G2 patients flowed to the low-risk subgroup (**Figure 4B**). What’s more, it was discovered that the expression of six prognostic genes had a gradual upward trend during the period from pathologic-T1 to TX (*P*<0.05), and the same trend was noted for the remaining five genes, except for CTHRC1, during the period from grade-1 to grade-X (*P*<0.05) (**Figure 4C**).

**Table 2:** Clinical characteristics of the two risk subgroups.

	Total	Risk		*Pvalue*
		high	low	
age(year)				
Mean (SD)	65.5 (±10.6)	65.3 (±10.9)	65.8 (±10.4)	0.73
gender				
FEMALE	124 (35.3%)	62 (35.4%)	62 (35.2%)	1
MALE	227 (64.7%)	113 (64.6%)	114 (64.8%)	
vital_status				
Alive	209 (59.5%)	89 (50.9%)	120 (68.2%)	0.001
Dead	142 (40.5%)	86 (49.1%)	56 (31.8%)	
OS(Months)				
Mean (SD)	612.0 (±548.5)	533.9 (±458.2)	689.6 (±617.0)	0.029
pathologic_M				
M0	313 (89.2%)	151 (86.3%)	162 (92.0%)	0.18
M1	22 (6.3%)	13 (7.4%)	9 (5.1%)	
MX	16 (4.6%)	11 (6.3%)	5 (2.8%)	
pathologic_N				
N0	103 (29.4%)	46 (26.4%)	57 (32.4%)	0.26
N1	95 (27.1%)	45 (25.9%)	50 (28.4%)	
N2	72 (20.6%)	36 (20.7%)	36 (20.5%)	
N3	71 (20.3%)	40 (23.0%)	31 (17.6%)	
NX	9 (2.6%)	7 (4.0%)	2 (1.1%)	
pathologic_T				
T1	18 (5.1%)	3 (1.7%)	15 (8.5%)	0.007
T2	74 (21.1%)	39 (22.3%)	35 (19.9%)	
T3	161 (45.9%)	77 (44.0%)	84 (47.7%)	
T4	94 (26.8%)	52 (29.7%)	42 (23.9%)	
TX	4 (1.1%)	4 (2.3%)	0 (0.0%)	
pathologic_stage				
Stage I	48 (14.2%)	19 (11.4%)	29 (16.9%)	0.54
Stage II	109 (32.2%)	54 (32.5%)	55 (32.0%)	
Stage III	147 (43.5%)	75 (45.2%)	72 (41.9%)	
Stage IV	34 (10.1%)	18 (10.8%)	16 (9.3%)	
Grade				
G1	9 (2.6%)	5 (2.9%)	4 (2.3%)	0.017
G2	126 (35.9%)	49 (28.0%)	77 (43.8%)	
G3	207 (59.0%)	117 (66.9%)	90 (51.1%)	
GX	9 (2.6%)	4 (2.3%)	5 (2.8%)	

Red value means p<0.05.

### 3.5 An effective nomogram was created in GC

**Figure 5:**
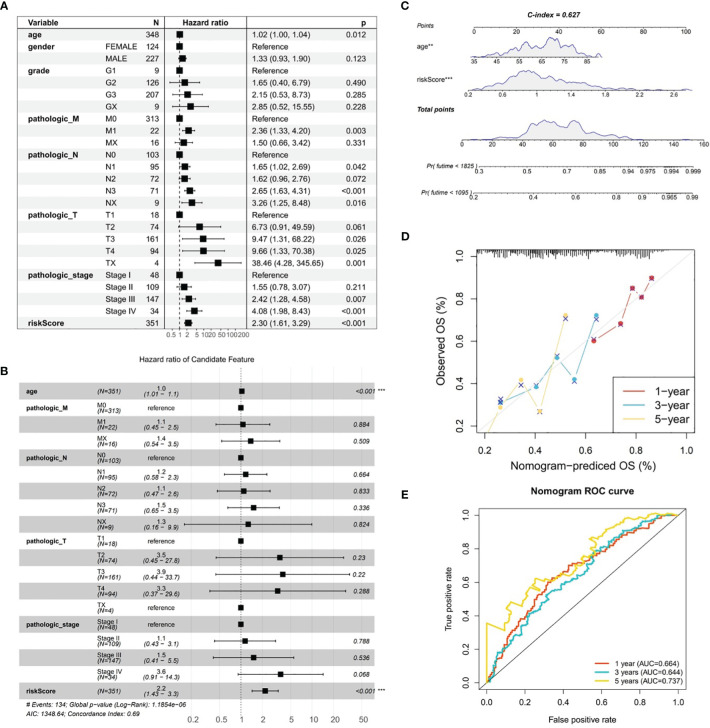
Analysis of gastric cancer in the TCGA-GC dataset combining risk scores and traditional clinical features. **(A)** Univariate Cox analysis of risk scores and traditional clinical features associated with overall survival in gastric cancer, **(B)** Independent prognostic indicators for predicting the prognosis of gastric cancer patients obtained through PH hypothesis testing and multivariate Cox analysis, **(C)** Nomogram constructed by combining risk scores and age for predicting the prognosis of gastric cancer, **(D)** Calibration curve of the nomogram, **(E)** ROC curve of the nomogram.

By combining conventional clinical characteristics with risk score in TCGA-GC dataset, univariate Cox analysis manifested that risk score, age, pathologic-M, pathologic-N, pathologic-T, and pathologic-stage were all markedly associated with OS in GC (*P*<0.05) (**Figure 5A**). On the basis of PH hypothesis test and multivariate Cox analysis, risk score and age were detected as independent prognostic indicators for predicting the prognosis of GC patients (**Figure 5B**). Subsequently, a nomogram integrating risk score and age for predicting GC prognosis was constructed (**Figure 5C**). The calibration curve suggested that the predicted and actual values of nomogram were roughly the same (**Figure 5D**). Meanwhile, AUC values at 1-, 3-, and 5-years were 0.664, 0.644, and 0.737, correspondingly (**Figure 5E**). In summary, the nomogram constructed in accordance with risk score and age had significant predictive value for predicting GC survival with higher predictive accuracy than risk model.

### 3.6 Differences in biological pathways between two risk subgroups were clarified

**Figure 6:**
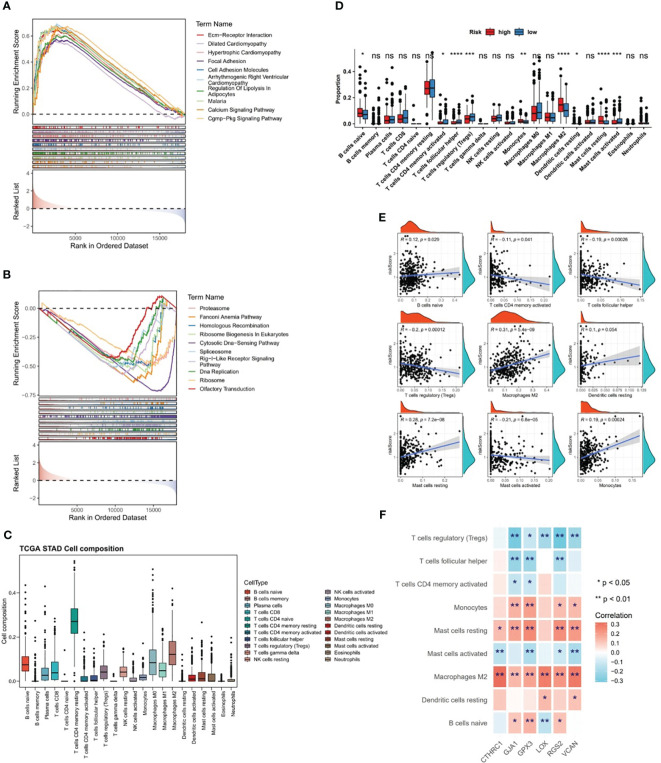
Differences in biological pathways and immune characteristics between two risk subgroups in the TCGA-GC dataset. **(A, B)** Gene Set Enrichment Analysis (GSEA) for the high-risk subgroup **(A)** and the low-risk subgroup **(B)**, **(C, D)** Analysis of different infiltration levels of immune cells between the two risk subgroups using the CIBERSORT program, **(E)** Correlation study between risk scores and different immune cells, **(F)** Correlation study between prognostic genes and different immune cells. * represent p<0.05, ** represent p<0.01, *** represent p<0.001, **** represent p<0.0001, ns represent no significant different.

The GSEA was conducted in TCGA-GC dataset to determine the most markedly enriched pathways between two risk subgroups. It was noted that genes from high-risk patients were markedly enriched in the “cell adhesion molecules”, “ECM-receptor interaction”, “cGMP-PKG signaling pathway”, “Calcium signaling pathway pathways”, etc (**Figure 6A**). However, genes in low-risk subgroup were markedly enriched in the following pathways, namely, “RIG-I-like receptor signaling pathway”, “cell cycle”, “oxidative phosphorylation pathway”, etc (**Figure 6B**). These analyses suggested that risk score was highly correlated with these biological pathways, providing insight valuable for understanding the potential molecular mechanisms of GC.

### 3.7 Risk score was associated with immunological features in GC

The CIBERSORT program was adopted to estimate the score of immune cells between the two risk subgroups, and the results revealed that resting-memory CD4^+^ T cells, M0 macrophages, M1 macrophages, and M2 macrophages were more enriched in samples of TCGA-GC dataset (**Figure 6C**). Further, it was noted that in case of statistical differences (*P*<0.05), the infiltration levels of naive B cells, M2 macrophages, monocytes, resting dendritic cells, and resting mast cells were higher in GC samples from high-risk subgroups, whereas the infiltration levels of activated-memory CD4^+^ T cells, follicular helper T cells, regulatory T cells (Tregs), and activated mast cells were higher in GC samples from low-risk subgroups (**Figure 6D**). The strongest positive correlation (cor=0.31 and *P*<0.001) between risk score and M2 macrophages was observed in the correlation study (**Figure 6E**). Simultaneously, it was also clear that there was significant correlations between prognostic genes and differential immune cells, in which the strongest positive association was found between CTHRC1 and M2 macrophages (cor=0.32 and *P*<0.001), and the strongest negative association was found between RGS2 and Tregs (cor=-0.31 and *P*<0.001) (**Figure 6F**).

**Figure 7:**
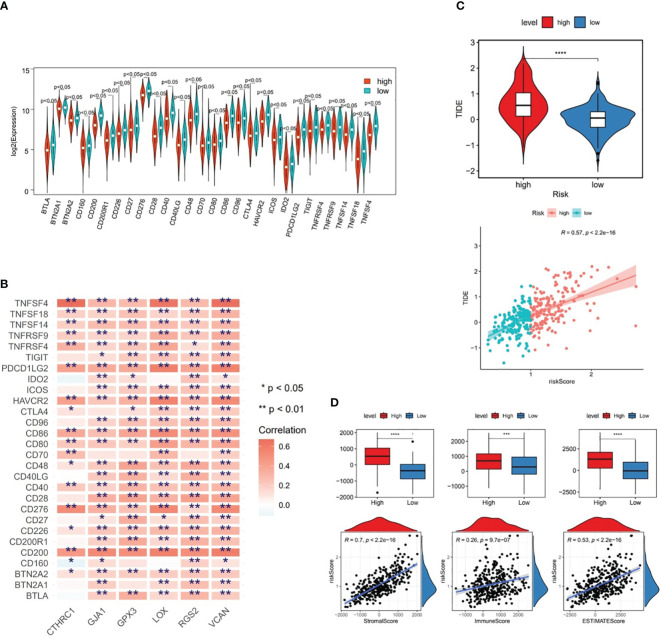
Differences in immune cell infiltration between two risk subgroups in the TCGA-GC dataset. **(A)** Immune cell gene (ICG) levels between the two risk subgroups, **(B)** Correlation analysis between six prognostic genes and immune cell genes, **(C)** TIDE scores between the two risk subgroups, **(D)** Immune score, stromal score, and ESTIMATE score between the two risk subgroups. * represent p<0.05, ** represent p<0.01, *** represent p<0.001, **** represent p<0.0001.

From **Figure 7A**, it could be observed that there were differences in 28 ICGs (BTLA, BTN2A1, BTN2A2, etc.) levels between the two risk subgroups, and all of these 28 ICGs levels were lower in high-risk subgroup compared to the low-risk group. Besides, there were multiple positive correlations between six prognostic genes and these 28 ICGs, among which TNFSF4 and CD200 were more strongly correlated with prognostic genes (**Figure 7B**). Further analyses demonstrated a marked difference in TIDE score between two risk subgroups and a notable positive association between TIDE score and risk score (cor=0.57 and *P*<0.001), predicting a better outcome for immunotherapy in low-risk patients (**Figure 7C**). Ultimately, ESTIMATE algorithm demonstrated higher immune, stromal, and ESTIMATE scores (*P*<0.001) in high-risk subgroup compared to the low-risk subgroup (**Figure 7D**), in other words, there was marked positive correlations between them and the risk score (stromal score: cor=0.7, immune score: cor=0.26, ESTIMATE score: cor=0.53; all *P*<0.001) (**Figure 7D**).

### 3.8 The relationship of risk score with chemotherapy agents, MSI, TMB, and CNV

**Figure 8:**
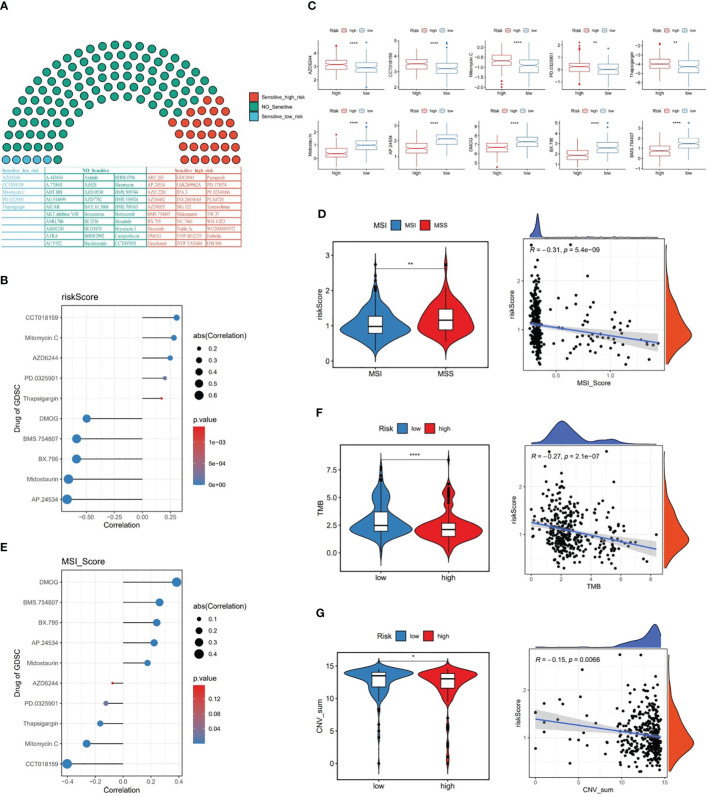
The relationship between risk score and chemotherapy drugs, MSI, TMB, and CNV. **(A)** Likelihood of sensitivity to different chemotherapy drugs, **(B)** Correlation between risk score and chemotherapy drugs, **(C)** Chemotherapy drug scores between the two risk subgroups, **(D)** MSI scores between the two risk subgroups and the correlation between MSI score and risk score, **(E)** Correlation between MSI score and chemotherapy drugs, **(F)** TMB scores between the two risk subgroups and the correlation between TMB score and risk score, **(G)** CNV scores between the two risk subgroups and the correlation between CNV score and risk score. * represent p<0.05, ** represent p<0.01, **** represent p<0.0001.

Among 138 chemotherapy agents, five agents (AZD6244, CCT018159, Mitomycin.C, etc.) belonged to sensitive low-risk group and 30 agents (AP.24534, Midostaurin, etc.) belonged to sensitive high risk group (**Figures 8A, B**). Of these, CCT018159 was markedly positively associated with risk score, as well as DMOG, BMS.754807, BX.795, Midostaurin, and AP.24534 were significantly negatively linked to risk score (*P*<0.05 and |cor|>0.3) (**Figure 8C**). These findings could provide a reliable reference for clinical treatment. Subsequently, with respect to the MSI analysis, patients in MSS group had a higher risk score than those in MSI group, and MSI score was markedly negatively linked to risk score (*P*<0.001 and cor=-0.31) (**Figure 8D**). Interestingly, it was noticed that the chemotherapeutic agent DMOG was significantly positively associated with MSI score, while the chemotherapeutic agent CCT018159 was significantly negatively associated with MSI score (*P*<0.05 and |cor|>0.3) (**Figure 8E**).

Further, our data revealed that TMB and CNV were markedly lower in high-risk patients than in low-risk patients, and risk score was remarkably negatively linked to TMB and CNV in GC (**Figures 8F, G**).

### 3.9 Stromal cells were identified as key cells

**Figure 9:**
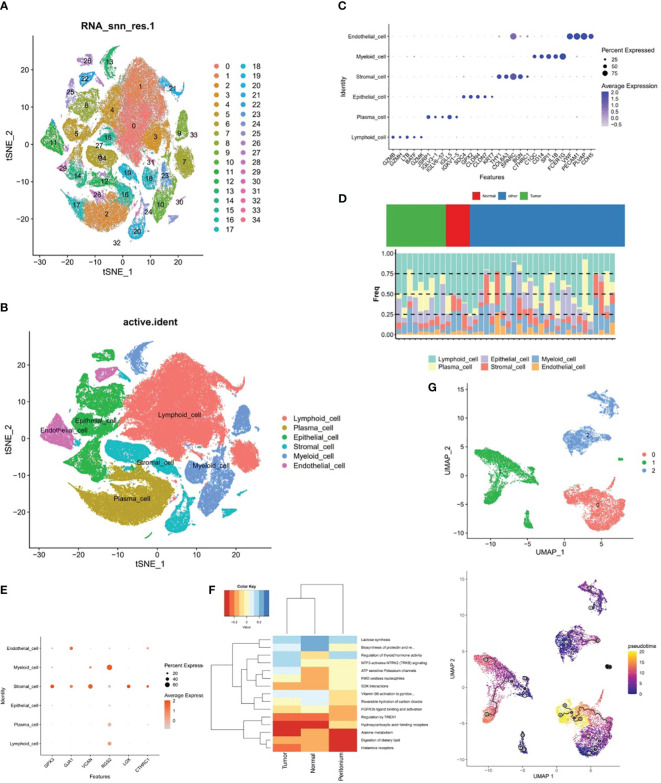
The relationship between stromal cells and prognostic genes. **(A, B)** Cell clusters in the GSE183904 dataset, **(C)** Significant marker genes corresponding to each of the six cell subsets. **(D)** Proportions of the six cell subsets, **(E)** Expression differences of six prognostic genes in six different cell subsets, **(F)** ReactomeGSA enrichment analysis of stromal cells in different tissues, **(G)** Pseudo-time analysis of three stromal cell subclasses.

In the samples of GSE183904 dataset, totally 130,770 cells and 25,504 genes were identified after QC for downstream analysis. After PCA dimensional reduction and unsupervised cluster analysis, 35 distinct cell clusters were identified (**Figure 9A**). Subsequently, cell annotation yielded six cell subpopulations, namely, lymphoid cell, epithelial cell, plasma cell, myeloid cell, stromal cell, and endothelial cell (**Figure 9B**). Meanwhile, **Figure 9C** revealed that the expression of the respective corresponding significant marker genes was higher in six cell subpopulations, for example, PECAM1, PLVAP, VWF, and CDH5 were highly expressed in endothelial cell, as well as FCER1G and SPARC had higher expression in myeloid cell and stromal cell, respectively. Besides, the proportion of these six cell subpopulations could be observed for the sample in GSE183904 dataset from **Figure 9D**, where lymphoid cell content was highest in all samples.

The expression discrepancies of the six prognostic genes were analyzed in six different cell subpopulations to further explore the expression of these genes at the cellular level. As demonstrated in **Figure 9E** and **Supplementary Figure 2**, the cells with more expression of the prognostic genes were stromal cell, myeloid cell, and endothelial cell, and all six genes were expressed in stromal cell, so stromal cell was employed as the key cell for subsequent analyses in this study. ReactomeGSA enrichment analysis demonstrated that stromal cells were primarily engaged in “ATP sensitive potassium channels”, “FMO oxidizes nucleophiles”, “regulation of thyroid hormone activity”, etc (**Figure 9F**). Further, stromal cells was divided into three subclasses (stromal C1, stromal C2, and stromal C3), and it was noted that a relatively complete developmental trajectory existed for stromal C1 in pseudo-time analysis (**Figure 9G**).

### 3.10 Chromosomal localization, subcellular localization, and potential regulatory analyses were completed

**Figure 10:**
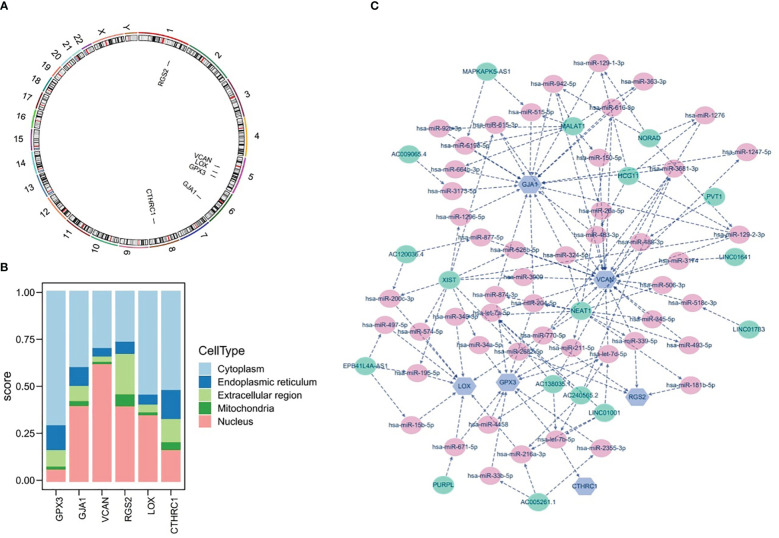
Chromosome Localization, subcellular Localization, and potential regulatory analysis. **(A)** Chromosome localization analysis of six prognostic genes, **(B)** subcellular localization of six prognostic genes, **(C)** lncRNA-miRNA-mRNA network of six prognostic genes.

The results of chromosomal localization analysis indicated that GPX3, LOX, and VCAN were all located in chromosomes 5, as well as RGS2, GJA1, and CTHRC1 in chromosomes 1, 6, and 8, respectively (**Figure 10A**). Meanwhile, the six prognostic genes were entered into the mRNALocater database to analyze their subcellular localisation, and the results revealed that GPX3, LOX, and CTHRC1 were preferably expressed in cytoplasm, whereas GJA1, VCAN, and RGS2 were preferably expressed in nucleus (**Figure 10B**).

Furthermore, the potential regulatory mechanisms of prognostic genes were elucidated by constructing a lncRNA-miRNA-mRNA network, as demonstrated in **Figure 10C**. It could be observed that the lncRNA-miRNA-mRNA network included 73 nodes (6 mRNA, 50 miRNA, and 17 lncRNA) and 133 edges, as well as the multiple relationship pairs were found in network. Obviously, lncRNAs (LINC01001, AC138035.1, and AC240565.2) could simultaneously regulate prognostic genes (VCAN, CTHRC1, and GPX3) via both hsa-let-7 family members (hsa-let-7a-5p, hsa-let-7b-5p, and hsa-let-7d-5p), as well as hsa-miR-200c-3p was identified as a regulator of LOX.

### 3.11 Experimental verification of prognostic genes expression in GC

**Figure 11:**
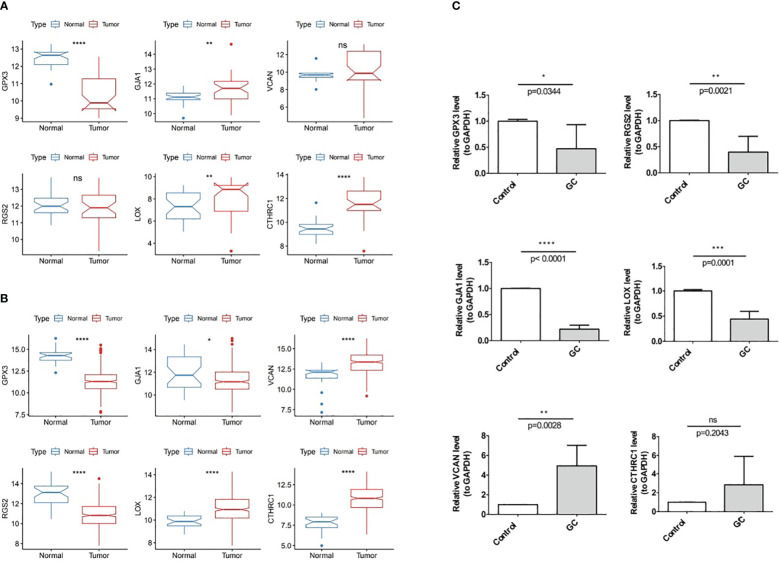
Experimental validation of prognostic gene Expression in GC, **(A)** Expression of six prognostic genes in the TCGA-GC dataset, **(B)** Expression of six prognostic genes in the GSE13911 dataset, **(C)** Expression of six prognostic genes in GC tumor tissues and adjacent normal tissues. * represent p<0.05, ** represent p<0.01, *** represent p<0.001, **** represent p<0.0001, ns represent no significant different.

In TCGA-GC dataset, the expression of the GJA1, VCAN, LOX, and CTHRC1 was markedly higher in GC samples than in normal samples, whereas GPX3 and RGS2 were markedly lower in the GC samples (**Figure 11A**). Besides, the expression of six prognostic genes was validated in GSE13911 dataset, and the results were presented in **Figure 11B**. Except for GJA1, the expression trends of the remaining five genes were consistent with the TCGA-GC dataset, and the expression of GPX3, LOX, and CTHRC1 had markedly difference between GC and normal samples (*P*<0.05).

With the purpose of verifying demonstrate the expression of prognostic genes in GC samples, qRT-PCR was performed on the GC tumor and the paraneoplastic normal tissues. As shown in **Figure 11C**, RGS2, GJA1, GPX3, and LOX were less expressed in gastric cancer tissues than in paracancerous tissues (*P*<0.05). The expression trend of VCAN was opposite (*P*<0.05), while CTHRC1 had no difference in expression between GC and paracancerous samples (*P*>0.05).

## 4 Discussion

Gastric cancer is a common malignant tumor of the digestive tract worldwide, and its pathogenesis involves various factors. In recent years, with the development of molecular biology, the role of mitochondrial function and macrophage polarization in gastric cancer has gradually attracted attention. Current studies have shown that mitochondrial function is closely related to the progression of gastric cancer (43), while macrophage polarization also plays an important role in the immune microenvironment of gastric cancer (44). However, the relationship between mitochondrial function and macrophage polarization in gastric cancer and the underlying genetic regulatory mechanisms remain unclear. This study aims to identify genes related to mitochondrial function and macrophage polarization in gastric cancer through bioinformatics analysis and biological sample validation, providing new ideas for the diagnosis and treatment of gastric cancer.

In this study, we first collected gene expression data from gastric cancer patients and screened out genes related to mitochondrial function and macrophage polarization using bioinformatics methods. We selected data of 375 cases of gastric cancer and 32 adjacent tissues from the TCGA dataset, as well as data from the GSE183904 dataset with complete single-cell sequencing data to identify related differentially expressed genes and establish the model. We also selected the larger sample-sized GSE15459 dataset and GSE13911 dataset for validation. To avoid potential selection bias, we used the data of all patients in the datasets for analysis. Then, we used bioinformatics tools to perform functional annotation and pathway analysis on these genes to reveal their possible mechanisms in gastric cancer. Through analysis, we identified a group of genes closely related to mitochondrial function and macrophage polarization in gastric cancer. These genes are mainly involved in biological processes such as energy metabolism, oxidative stress, and immune response. In addition, we also found that these genes are closely related to the prognosis of gastric cancer, suggesting that they may be potential targets for gastric cancer treatment.

Glutathione peroxidase 3 (GPX3), the only known extracellular glycosylated enzyme in the glutathione peroxidase family, is a protein that contains a selenocysteine residue. It plays an important role in cellular defense mechanisms by resisting stress signals and scavenging reactive oxygen species, thereby maintaining the genetic integrity of cells (45). GPX3 can induce mitochondrial-related apoptosis through the AMPK/ERK1/2 pathway (46). GPX3 is also correlated with macrophage infiltration in tumors (47). In various tumors, the GPX3 promoter is hypermethylated or its allele is lost, leading to low expression (48, 49). Low expression of GPX3 is closely related to the occurrence, development, and prognosis of tumors such as gastric cancer (50).

Regulator of G protein signalling 2 (RGS2) is involved in cell cycle, transmembrane receptor protein tyrosine kinase signaling pathway, and regulation of G protein-coupled receptor protein signaling pathway, with a negative regulatory function in signal transduction (51). Studies have shown that the RGS2 gene also plays a certain role in cancer. In gastric cancer, the RGS2 gene is considered a new tumor biomarker. Fatty acid metabolism is related to the changes in the immune microenvironment of gastric cancer, and the RGS2 gene may participate in this process by regulating the G protein signaling pathway (52). Additionally, the RGS2 gene exhibits abnormal expression in prostate cancer. During the progression of prostate cancer, the downregulation of RGS2 expression is associated with hypoxia and is related to the regulation and influence of tumor phenotypes (53).

Versican, the protein encoded by the VCAN gene, is an important extracellular matrix protein involved in biological processes such as cell adhesion, migration, proliferation, and signaling. Research has shown that the VCAN gene is negatively regulated by methylation, leading to its high expression in cancer tissues (54). In gastric cancer, VCAN is overexpressed and can predict the response to adjuvant chemotherapy, adjuvant radiotherapy and immunotherapy (55).

The CTHRC1 gene encodes collagen triple helix repeat protein 1, which plays an important role in various biological processes, including inhibiting collagen deposition, promoting cell migration, and accelerating vascular repair. In recent years, the role of CTHRC1 in cancer research has gradually emerged, especially in gastric cancer, hepatocellular carcinoma, colorectal cancer, esophageal cancer, and other cancers (56). The expression of CTHRC1 protein in gastric cancer tissues is significantly higher than that in adjacent tissues, and there is a certain correlation between the expression of CTHRC1 protein and the prognosis of gastric cancer patients. CTHRC1 increases the expression of CXCR4 by up-regulating the expression of HIF-1α, ultimately promoting cell migration and invasion (57). In colon cancer, CTHRC1 remodels infiltrating macrophages through interaction with TGF-β receptors, promoting liver metastasis of colorectal cancer cells (58).

The gene for Gap Junction Alpha-1 (GJA1), also known as Connexin43 (Cx43), is a key gap junction protein necessary for the propagation of action potentials between adjacent cells (59). The GJA1 gene also plays a role in cancer. In breast cancer, the expression of GJA1 is related to tumor subtype (60). In colorectal cancer, the loss of GJA1 expression is positively correlated with patient metastasis and poor prognosis. Overexpression of GJA1 can inhibit the progression of colorectal cancer and enhance cancer cell sensitivity to 5-fluorouracil (5-FU) (61). The function of the GJA1 gene in gastric cancer is still unclear. Some studies have shown that the expression level of GJA1 protein is low in gastric cancer tissue, and its low expression is associated with the progression and poor prognosis of gastric cancer (62), which is consistent with our analysis results but inconsistent with the results of the TCGA database. This suggests that the role of the GJA1 gene in the occurrence and development of gastric cancer may be complex. Currently, the specific mechanism of GJA1 in gastric cancer still requires further investigation to provide new ideas and methods for the diagnosis and treatment of gastric cancer.

LOX is a copper-dependent monoamine oxidase that participates in the covalent cross-linking of collagen and elastin in the extracellular matrix, thereby maintaining the normal structure and function of the extracellular matrix (63). LOX can affect VEGF induction, HIF-1α activation, and other mechanisms, playing an important role in the occurrence, development, invasion, and metastasis of various tumor (64). In gastric cancer, the downregulation of LOX expression can downregulate the expression of MMP-2 and MMP-9 in cancer cells (65). Literature shows that the expression level of LOX in gastric cancer is usually high, which is consistent with the results of TCGA and GEO databases (66). However, our verification results may be limited by the sample size and show opposite results, which can be further verified with more samples in the future.

This study revealed the potential mechanism of mitochondrial function and macrophage polarization-related genes in gastric cancer through bioinformatics analysis, and verified the expression of these genes in gastric cancer tissues by qRT-PCR, providing new ideas for the diagnosis and treatment of gastric cancer. Gene diagnosis is playing an increasingly important role in clinical work. Currently, samples from patients after surgery are often subject to genetic analysis. Therefore, in future work, it is highly feasible to apply the nomogram composed of these genes for clinical diagnosis, prognosis determination and clinical decision-making. The target genes screened in this study have not yet been functionally validated at the cellular level. These genes may not only function in tumor cells but also play important roles in stromal cells and affect the behavior of tumor cells. Stromal cells in tumors may impact aspects such as tumor occurrence, development, metastasis, and treatment response through means like growth factor signal transduction, influencing the function of immune cells in the tumor microenvironment, and providing nutrients for tumor cells. In the future, we will further validate the functions of these genes through cellular and animal experiments, focusing on exploring their mechanisms in mitochondrial function and macrophage polarization in gastric cancer, in order to more comprehensively understand the pathogenesis and treatment of gastric cancer.

## Data availability statement

The original contributions presented in the study are included in the article/**Supplementary Material**. Further inquiries can be directed to the corresponding authors.

## Ethics statement

The studies involving humans were approved by Gusu School, Nanjing Medical Universtiy. The studies were conducted in accordance with the local legislation and institutional requirements. The participants provided their written informed consent to participate in this study.

## Author contributions

YZ: Conceptualization, Writing – original draft, Writing – review & editing. JC: Data curation, Writing – original draft. ZY: Formal analysis, Writing – original draft. HZ: Investigation, Writing – original draft. JY: Methodology, Writing – original draft. XT: Software, Writing – original draft. XG: Project administration, Writing – review & editing.

## Supplementary material

Supplementary Figure 1 Genes significantly associated with prognosis obtained through univariate Cox regression analysis and PH assumption testing.

Supplementary Figure 2 Expression differences of six prognostic genes across six different cell subpopulations.

Supplementary Table 1 1,136 Mitochondrial-Related Genes (MRGs).

Supplementary Table 2 35 Macrophage Polarization-Related Genes (MPRGs).
