# Supplementary material for: Construction and validation of prognostic signatures related to mitochondria and macrophage polarization in gastric cancer
Source: Front Oncol. 2024 Jul 26;14:1433874. doi: 10.3389/fonc.2024.1433874 (PMC11310369; doi:10.3389/fonc.2024.1433874)
Supplement: Supplementary Figure 1 — Genes significantly associated with prognosis obtained through univariate Cox regression analysis and PH assumption testing. [file DataSheet_1.pdf]

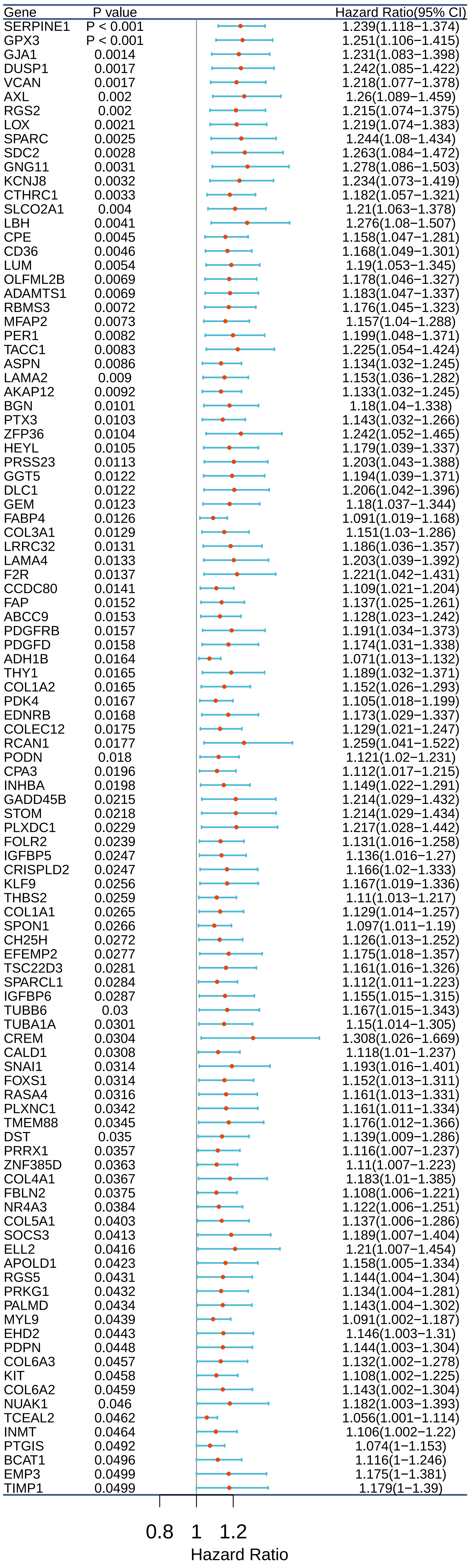
Gene P value Hazard Ratio(95% CI)
SERPINE1 P < 0.001 1.239(1.118−1.374)
GPX3 P < 0.001 1.251(1.106−1.415)
GJA1 0.0014 1.231(1.083−1.398)
DUSP1 0.0017 1.242(1.085−1.422)
VCAN 0.0017 1.218(1.077−1.378)
AXL 0.002 1.26(1.089−1.459)
RGS2 0.002 1.215(1.074−1.375)
LOX 0.0021 1.219(1.074−1.383)
SPARC 0.0025 1.244(1.08−1.434)
SDC2 0.0028 1.263(1.084−1.472)
GNG11 0.0031 1.278(1.086−1.503)
KCNJ8 0.0032 1.234(1.073−1.419)
CTHRC1 0.0033 1.182(1.057−1.321)
SLCO2A1 0.004 1.21(1.063−1.378)
LBH 0.0041 1.276(1.08−1.507)
CPE 0.0045 1.158(1.047−1.281)
CD36 0.0046 1.168(1.049−1.301)
LUM 0.0054 1.19(1.053−1.345)
OLFML2B 0.0069 1.178(1.046−1.327)
ADAMTS1 0.0069 1.183(1.047−1.337)
RBMS3 0.0072 1.176(1.045−1.323)
MFAP2 0.0073 1.157(1.04−1.288)
PER1 0.0082 1.199(1.048−1.371)
TACC1 0.0083 1.225(1.054−1.424)
ASPN 0.0086 1.134(1.032−1.245)
LAMA2 0.009 1.153(1.036−1.282)
AKAP12 0.0092 1.133(1.032−1.245)
BGN 0.0101 1.18(1.04−1.338)
PTX3 0.0103 1.143(1.032−1.266)
ZFP36 0.0104 1.242(1.052−1.465)
HEYL 0.0105 1.179(1.039−1.337)
PRSS23 0.0113 1.203(1.043−1.388)
GGT5 0.0122 1.194(1.039−1.371)
DLC1 0.0122 1.206(1.042−1.396)
GEM 0.0123 1.18(1.037−1.344)
FABP4 0.0126 1.091(1.019−1.168)
COL3A1 0.0129 1.151(1.03−1.286)
LRRC32 0.0131 1.186(1.036−1.357)
LAMA4 0.0133 1.203(1.039−1.392)
F2R 0.0137 1.221(1.042−1.431)
CCDC80 0.0141 1.109(1.021−1.204)
FAP 0.0152 1.137(1.025−1.261)
ABCC9 0.0153 1.128(1.023−1.242)
PDGFRB 0.0157 1.191(1.034−1.373)
PDGFD 0.0158 1.174(1.031−1.338)
ADH1B 0.0164 1.071(1.013−1.132)
THY1 0.0165 1.189(1.032−1.371)
COL1A2 0.0165 1.152(1.026−1.293)
PDK4 0.0167 1.105(1.018−1.199)
EDNRB 0.0168 1.173(1.029−1.337)
COLEC12 0.0175 1.129(1.021−1.247)
RCAN1 0.0177 1.259(1.041−1.522)
PODN 0.018 1.121(1.02−1.231)
CPA3 0.0196 1.112(1.017−1.215)
INHBA 0.0198 1.149(1.022−1.291)
GADD45B 0.0215 1.214(1.029−1.432)
STOM 0.0218 1.214(1.029−1.434)
PLXDC1 0.0229 1.217(1.028−1.442)
FOLR2 0.0239 1.131(1.016−1.258)
IGFBP5 0.0247 1.136(1.016−1.27)
CRISPLD2 0.0247 1.166(1.02−1.333)
KLF9 0.0256 1.167(1.019−1.336)
THBS2 0.0259 1.11(1.013−1.217)
COL1A1 0.0265 1.129(1.014−1.257)
SPON1 0.0266 1.097(1.011−1.19)
CH25H 0.0272 1.126(1.013−1.252)
EFEMP2 0.0277 1.175(1.018−1.357)
TSC22D3 0.0281 1.161(1.016−1.326)
SPARCL1 0.0284 1.112(1.011−1.223)
IGFBP6 0.0287 1.155(1.015−1.315)
TUBB6 0.03 1.167(1.015−1.343)
TUBA1A 0.0301 1.15(1.014−1.305)
CREM 0.0304 1.308(1.026−1.669)
CALD1 0.0308 1.118(1.01−1.237)
SNAI1 0.0314 1.193(1.016−1.401)
FOXS1 0.0314 1.152(1.013−1.311)
RASA4 0.0316 1.161(1.013−1.331)
PLXNC1 0.0342 1.161(1.011−1.334)
TMEM88 0.0345 1.176(1.012−1.366)
DST 0.035 1.139(1.009−1.286)
PRRX1 0.0357 1.116(1.007−1.237)
ZNF385D 0.0363 1.11(1.007−1.223)
COL4A1 0.0367 1.183(1.01−1.385)
FBLN2 0.0375 1.108(1.006−1.221)
NR4A3 0.0384 1.122(1.006−1.251)
COL5A1 0.0403 1.137(1.006−1.286)
SOCS3 0.0413 1.189(1.007−1.404)
ELL2 0.0416 1.21(1.007−1.454)
APOLD1 0.0423 1.158(1.005−1.334)
RGS5 0.0431 1.144(1.004−1.304)
PRKG1 0.0432 1.134(1.004−1.281)
PALMD 0.0434 1.143(1.004−1.302)
MYL9 0.0439 1.091(1.002−1.187)
EHD2 0.0443 1.146(1.003−1.31)
PDPN 0.0448 1.144(1.003−1.304)
COL6A3 0.0457 1.132(1.002−1.278)
KIT 0.0458 1.108(1.002−1.225)
COL6A2 0.0459 1.143(1.002−1.304)
NUAK1 0.046 1.182(1.003−1.393)
TCEAL2 0.0462 1.056(1.001−1.114)
INMT 0.0464 1.106(1.002−1.22)
PTGIS 0.0492 1.074(1−1.153)
BCAT1 0.0496 1.116(1−1.246)
EMP3 0.0499 1.175(1−1.381)
TIMP1 0.0499 1.179(1−1.39)
0.8
1
1.2
Hazard Ratio
