# Supplementary figures and images for: Construction and validation of prognostic signatures related to mitochondria and macrophage polarization in gastric cancer
Source: Front Oncol. 2024 Jul 26;14:1433874. doi: 10.3389/fonc.2024.1433874 (PMC11310369; doi:10.3389/fonc.2024.1433874)

**GPX3**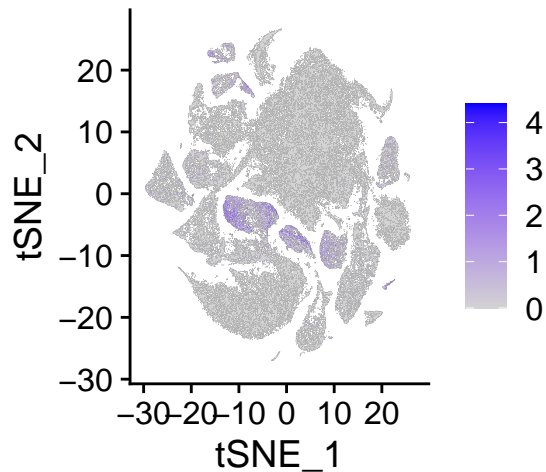**GJA1**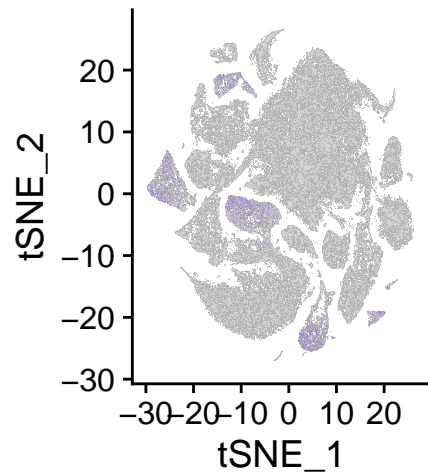**VCAN**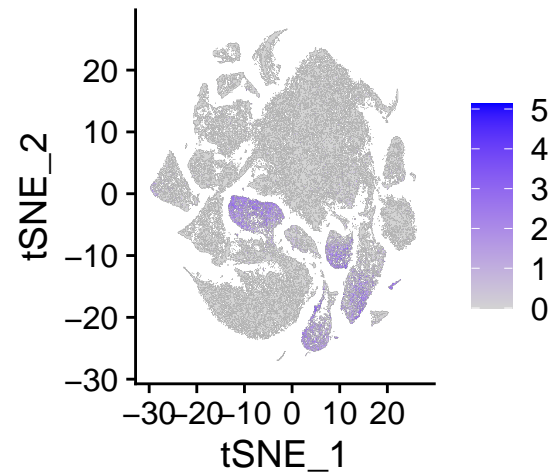**RGS2**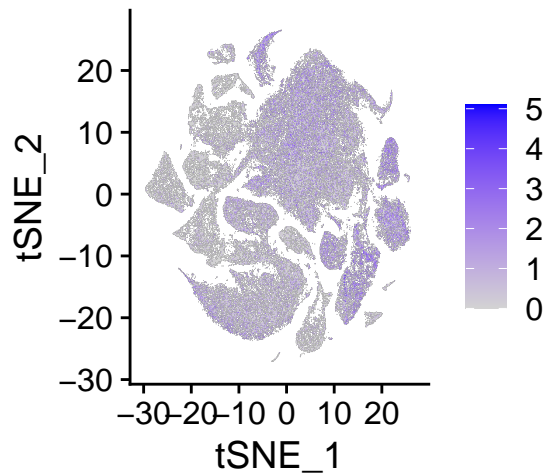**LOX**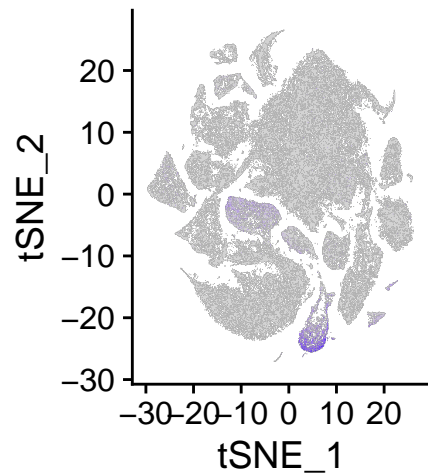**CTHRC1**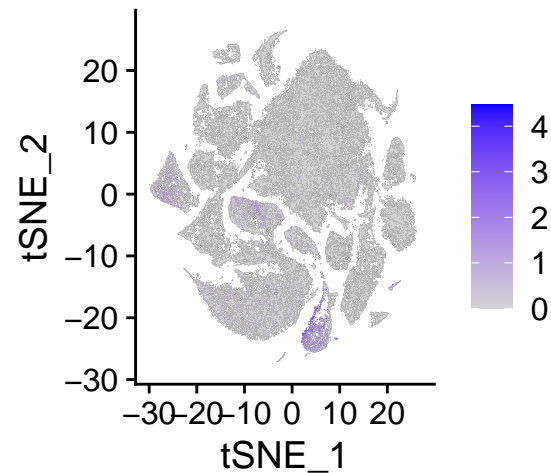

Supplement: Supplementary Figure 2 — Expression differences of six prognostic genes across six different cell subpopulations. [file DataSheet_2.pdf]
